# Identification of a reciprocal negative feedback loop between tau-modifying proteins MARK2 kinase and CBP acetyltransferase

**DOI:** 10.1016/j.jbc.2022.101977

**Published:** 2022-04-22

**Authors:** Zarin Tabassum, Jui-Heng Tseng, Camryn Isemann, Xu Tian, Youjun Chen, Laura E. Herring, Todd J. Cohen

**Affiliations:** 1 Department of Neurology, UNC Neuroscience Center, University of North Carolina at Chapel Hill, Chapel Hill, North Carolina, USA; 2 UNC Proteomics Core Facility, Department of Pharmacology, University of North Carolina, Chapel Hill, North Carolina, USA; 3 Department of Biochemistry and Biophysics, University of North Carolina, Chapel Hill, North Carolina, USA

**Keywords:** MARK2, CBP, tau, kinase, acetyltransferase, phosphorylation, acetylation, aggregation, neurodegeneration, AD, Alzheimer’s disease, CBP, CREB-binding protein, co-IP, coimmunoprecipitation, GAPDH, glyceraldehyde-3-phosphate dehydrogenase, MAP, MT-associated protein, MARK2, microtubule affinity-regulating kinase 2, MT, microtubule, MTBR, MT-binding region, NES, nuclear export signal, NFT, neurofibrillary tangle, PTM, posttranslational modification

## Abstract

The posttranslational regulation of the neuronal proteome is critical for brain homeostasis but becomes dysregulated in the aged or diseased brain, in which abnormal posttranslational modifications (PTMs) are frequently observed. While the full extent of modified substrates that comprise the “PTM-ome” are slowly emerging, how the upstream enzymes catalyzing these processes are regulated themselves is not well understood, particularly in the context of neurodegeneration. Here, we describe the reciprocal regulation of a kinase, the microtubule affinity-regulating kinase 2 (MARK2), and an acetyltransferase, CREB-binding protein (CBP), two enzymes known to extensively modify tau proteins in the progression of Alzheimer’s disease. We found that MARK2 negatively regulates CBP and, conversely, CBP directly acetylates and inhibits MARK2 kinase activity. These findings highlight a reciprocal negative feedback loop between a kinase and an acetyltransferase, which has implications for how PTM interplay is coordinated on substrates including tau. Our study suggests that PTM profiles occur through the posttranslational control of the master PTM remodeling enzymes themselves.

Distinct patterns of posttranslational modifications (PTMs) have emerged as a distinguishing and disease-relevant feature (1, 2, 3, 4, 5). Rather than individual PTMs mediating specific outputs, in many cases, a coordinated assembly of different PTMs targeting the same substrate can lead to major regulatory changes including altered electrostatic interactions between proteins, altered protein/enzyme activity, or altered biophysical properties of a substrate. Such dynamic PTM interplay is best exemplified by the regulation of histones, in which combined acetylation and phosphorylation can modify histone H3 N-terminal tails and thereby influence gene expression (6). More recent analysis in human Alzheimer’s disease (AD) brain showed more complex histone PTM dynamics, dependent on the affected brain regions and the severity of disease progression. For example, some histone PTMs generally increased with aging but become reduced in AD brain (*e.g.*, H4K16ac) (7, 8).

Beyond histones, the extent to which other substrates are coordinately regulated by PTMs in the brain is not well studied. One of the hallmark features of AD is the presence of neurofibrillary tangles (NFTs), composed of intracellular tau aggregates, that are extensively modified by phosphorylation, acetylation, and ubiquitination among other modifications (9, 10). In addition to tau, other pathological proteins implicated in various neurodegenerative diseases including α-synuclein (11), huntingtin (12, 13), and TDP-43 (14, 15) also undergo extensive PTM remodeling, indicating that this phenomenon is not limited to tau but is likely more widespread in the diseased brain. One can potentially explain these observations through shared pathogenic mechanisms that generate distinct PTM profiles as a consequence of aging, disease onset, or disease progression.

How such PTM profiles are choreographed is unclear, but prior studies on tau suggest a highly regulated PTM cascade. For example, tau kinases target specific Ser/Thr residues in tau that, in many cases, can prime additional nearby phosphorylation sites (16, 17, 18, 19, 20). In other instances, tau phosphorylation can inhibit the acetylation of nearby lysines (21, 22, 23). Finally, identical lysine residues on tau are subjected to multiple distinct PTMs including acetylation (24, 25), sumoylation (26, 27), methylation (28, 29, 30), or ubiquitination (31, 32). Therefore, complex PTM cascades may underlie tau’s functional output leading to changes in microtubule (MT) dynamics or tau aggregation. This implies that PTM profiles may initially be established by the remodeling enzymes that act upstream, whose regulation themselves now becomes a critical focal point in the context of the diseased brain.

Microtubule affinity-regulating kinases (MARKs) have become a topic of interest in AD due to their high affinity for tau, their ability to alter tau aggregation, and their potent regulation of tau-MT binding (33, 34, 35). Once active, MARK2 can phosphorylate tau at four residues within the MT binding region (MTBR) (Ser-262, Ser-293, Ser-324, and Ser-356) (36) and was shown to colocalize with tau in AD brain (37). Activation of MARK2 occurs through the phosphorylation of residue T208 within the activation loop and is coincident with local structural changes by which MARK2 switches from the inactive to active state (38). These findings led us to hypothesize that combinatorial PTMs may directly control MARK2 as a critical upstream event and thereby elicit widespread effects on downstream substrates including tau.

The well characterized acetyltransferase CREB-binding protein (CBP) is known to regulate histones and alter gene transcription, but more recently we and others demonstrated that MT-associated proteins (MAPs) including tau and related MAP2 and MAP4 family members are direct substrates of CBP acetyltransferase activity (24, 25, 39, 40, 41, 42). MAP family acetylation at specific lysine residues impairs MT stabilization and, at least in the context of tau, can promote its aberrant aggregation (24). Given that CBP and MARK2 have common downstream substrates including tau (43, 44), this prompted us to investigate their interplay and whether the activities of a kinase (MARK2) and an acetyltransferase (CBP) could potentially be coordinated. How PTM remodeling enzymes are regulated has implications for many neurodegenerative disorders characterized by dysfunctional “PTM-omes”. Here, we demonstrate that CBP and MARK2 engage in a reciprocal negative feedback loop that controls their levels and enzymatic activities.

## Results

### MARK2 modulates global lysine acetylation

**Figure 1:**
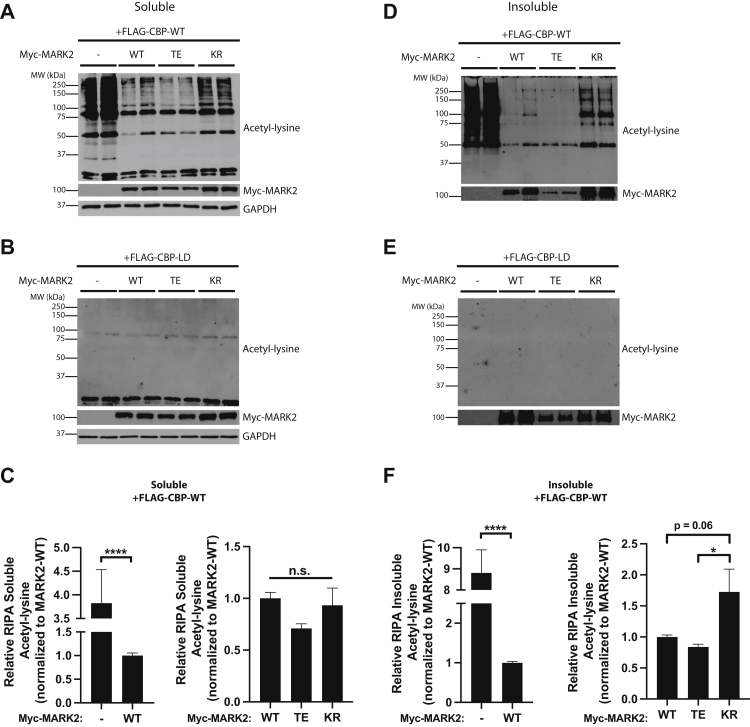
**MARK2 modulates CBP acetyltransferase activity.***A*–*C*, soluble RIPA-extracted fractions were examined from 293A cells transfected with WT or catalytically inactive CBP (LD) variants in parallel with either MARK2-WT, constitutively active MARK2 (T208E, MARK2-TE), or catalytically inactive MARK2 (K82R, MARK2-KR) and immunoblotted with a pan-acetyl-lysine antibody (*A* and *B*). The relative band intensities of the soluble acetyl-lysine immunoreactivity in the presence of CBP-WT were quantified and plotted in (*C*). The graphs represent changes in acetyl-lysine immunoreactivity observed in the presence of MARK2-WT compared to control, as well as relative differences observed among the MARK2 variants. Error bars indicate SEM. n = 8 biologically independent experiments. *p* value was assessed by either unpaired Student’s *t* test (*left panel*) or one-way ANOVA with Tukey’s post hoc test (*right panel*). n.s. *p* > 0.05; ∗∗∗∗ *p* < 0.0001. *D*–*F*, RIPA-insoluble cell pellets were extracted with urea buffer to isolate the aggregated protein fraction from the same transfection described above in (*A*) and samples were similarly immunoblotted with a pan-acetyl-lysine antibody (*D* and *E*). The relative band intensities of insoluble acetyl-lysine immunoreactivity in the presence of CBP-WT were quantified and plotted in (*F*) similar to (*C*) above. Error bars indicate SEM. n = 8 biologically independent experiments. *p* value was assessed by either unpaired Student’s *t* test (*left panel*) or one-way ANOVA with Tukey’s post hoc test (*right panel*). ∗*p* < 0.05; ∗∗∗∗*p* < 0.0001. CBP, CREB-binding protein; MARK2, microtubule affinity-regulating kinase 2.

Given that CBP acetylates tau on lysine residues within the MTBR, a region harboring KXGS motifs that are also targeted by MARK2, we suspected potential interplay among tau acetyltransferases and tau kinases, namely between CBP and MARK2. To test this possibility, we ectopically expressed both CBP and MARK2 in a human cell line (293A cells) and examined readouts of CBP acetyltransferase activity using a well-characterized pan-acetyl-lysine antibody that nondiscriminately detects lysine-acetylated substrates (Fig. 1). We examined acetylated lysine in the presence of three different MARK2 variants; WT MARK2, catalytically inactive MARK2 containing a K82R mutation (KR), and a constitutively active T208E mutant (TE) known to increase MARK2 activity ∼ four-fold (38, 45). Since CBP activity can promote protein aggregation that is recovered in biochemically insoluble fractions (46), lysates were sequentially fractionated using buffers of increasing extraction stringency, in which RIPA buffer was used to extract soluble proteins followed by a urea-based extraction of insoluble aggregated protein pellets. In this manner, we were able to examine both soluble (Fig. 1, *A*–*C*) and insoluble (Fig. 1, *D*–*F*) fractions by subsequent immunoblotting. In the soluble fractions, we observed significantly reduced CBP activity in the presence of MARK2-WT or active MARK2-TE, while the inactive MARK2-KR showed a trend toward reduced CBP inhibitory activity as determined by partly restored acetylated lysine levels (Fig. 1, *A* and *C*). We note that given its constitutive activation status, MARK-TE is consistently expressed at lower steady-state levels in cells compared to the less active WT and KR mutant (Fig. 1*A*). As a control, a similar analysis was performed using a catalytically inactive CBP-LD mutant (containing L1435A/D1436A mutations), which did not facilitate lysine acetylation (Fig. 1*B*).

Since CBP can promote generalized protein aggregation (46), we suspected that the effects of MARK2 on CBP activity would be more robust in the insoluble urea-extracted fractions containing aggregated proteins. Indeed, CBP generated a ∼ 50 to 250 kDa acetyl-lysine banding profile that was largely abrogated by either MARK2-WT or MARK2-TE, but not the inactive KR mutant (Fig. 1, *D* and *F*), while the inactive CBD-LD again had no impact on aggregation or protein acetylation (Fig. 1*E*). These data support MARK2 as a negative regulator of CBP acetyltransferase activity.

### MARK2 interacts with and regulates CBP levels

**Figure 2:**
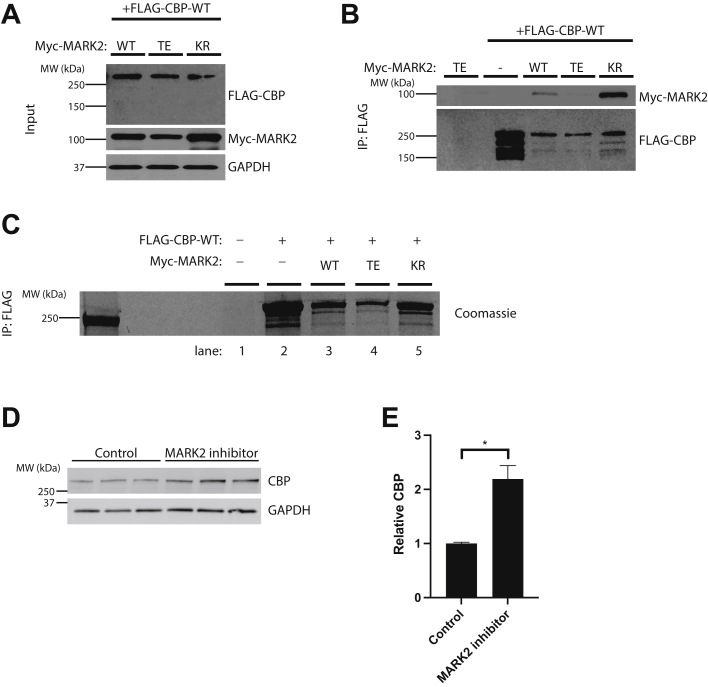
**CBP preferentially associates with inactive MARK2.***A* and *B*, coimmunoprecipitation (Co-IP) assays were performed to evaluate the binding of CBP to different MARK2 variants that show varying levels of kinase activity (MARK2-WT, MARK2-TE, MARK2-KR) in 293A cells by FLAG pull-down followed by immunoblotting to detect the total pool of myc-tagged MARK2 (Myc-MARK2). Input and IP samples are shown in (*A*) and (*B*) respectively. CBP preferentially interacts with the catalytically inactive MARK2 (MARK2-KR). *C*, Coomassie staining was used to detect immunoprecipitated FLAG-tagged CBP-WT in the absence (lane 1) or presence of different MARK2 variants (lanes 2–5) to illustrate a moderate reduction of CBP levels in the presence of active MARK2 (MARK2-WT and MARK2-TE). *D* and *E*, primary mouse cortical neurons at DIV14 were treated with or without 20 μM MARK2 inhibitor (39621) for 5 h. Neurons were then harvested and lysed, followed by immunoblotting with total CBP and GAPDH antibodies, and the increased CBP levels were quantified in (e). Error bars indicate SEM. n = 3 biologically independent experiments. *p* value was assessed by unpaired Student’s *t* test. ∗*p* < 0.05. CBP, CREB-binding protein; MARK2, microtubule affinity-regulating kinase 2.

We next examined a potential direct physical interaction between CBP and MARK2 through a series of coimmunoprecipitation (co-IP) assays in transfected cells. Myc-tagged MARK2 was coexpressed with FLAG-tagged CBP and subsequently processed by FLAG pull-downs followed by immunoblotting. The input revealed that all MARK2 variants were expressed, though as noted above, the active MARK-TE showed slightly reduced steady-state levels due to its heightened enzymatic activity (Fig. 2*A*). Interestingly, we observed a subtle but detectable interaction between CBP and MARK2-WT, while the strongest interaction occurred with MARK2-KR, suggesting preferential binding of CBP to inactive MARK2 (Fig. 2*B*). Consistent with preference for inactive MARK2, we were unable to detect CBP binding to the constitutively active MARK2-TE. Thus, CBP preferentially associates with inactive MARK2, suggesting the inactive MARK2 conformation is a more favorable binding partner and potential substrate for CBP.

To interrogate how MARK2 might regulate CBP, we considered MARK2-mediated phosphorylation of CBP and performed a series of cell-based assays, but we were unable to observe MARK2-dependent phosphorylation of CBP (Fig. S1). This result is not unexpected since CBP phosphorylation is thought to activate rather than suppress its acetyltransferase activity (47, 48). We therefore explored other mechanisms for how MARK2 might negatively regulate CBP. To do so, we immunoprecipitated total full-length CBP from cells coexpressing CBP and the MARK2 variants. However, to achieve more robust CBP detection sensitivity (CBP protein migrates at ∼ 300 kD), we employed a large-scale transfection strategy and immunoprecipitated a higher final yield of CBP followed by Coomassie gel staining. Using this total CBP detection method, we found that active MARK2, particularly the MARK2-TE variant, led to reduced total CBP protein levels compared to the less active MARK2-WT and the inactive MARK2-KR (Fig. 2*C*). To corroborate and extend the finding that MARK2 negatively regulates CBP levels, we treated primary mouse cortical neurons with a MARK2-specific pharmacological inhibitor (39621) and observed significantly increased CBP levels in response to MARK2 inhibition (Fig. 2, *D* and *E*), further supporting a negative regulatory mechanism in which MARK2 activity is required to maintain lower baseline CBP levels.

### CBP acetylates and impairs MARK2 kinase activity

**Figure 3:**
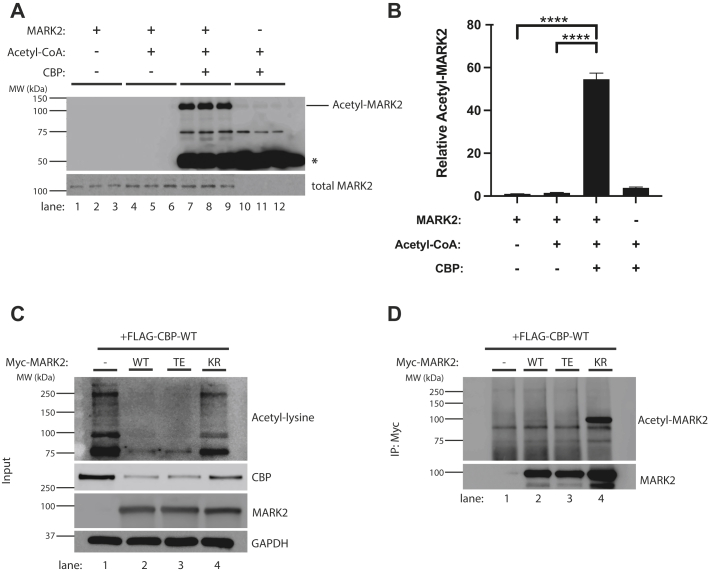
**MARK2 is subjected to CBP-mediated lysine acetylation.***A* and *B*, recombinant WT MARK2 containing a GST tag (MARK2-WT) was incubated with recombinant WT CBP catalytic domain in the presence or absence of the cofactor acetyl-CoA, where indicated, in acetylation buffer for 1.5 h at 37 °C. The *in vitro* acetylation reaction was terminated by the addition of 6X SDS sample buffer and analyzed by immunoblotting using an acetyl-lysine antibody. Acetylated GST-tagged MARK2 is specifically detected at ∼ 120 kD, and the asterisk indicates acetylated CBP, which is known to be generated *via* CBP auto-acetylation. Acetyl-MARK2 levels were quantified and plotted in (*B*). Error bars indicate SEM. n = 3 biologically independent experiments. *p* value was assessed by one-way ANOVA with Tukey’s post hoc test. ∗∗∗∗*p* < 0.0001. *C* and *D*, to confirm that MARK2 is acetylated in cells, a MARK2 immunoprecipitation was performed in 293A cells cotransfected with CBP and the various MARK2 variants. First, the presence of total CBP and MARK2 as inputs (*C*) were verified and the expected reduction in acetyl-lysine by active MARK2 was confirmed. Subsequently, the immunoprecipitated MARK2 fractions were blotted with the acetyl-lysine antibody to detect acetylated MARK2 (*D*) (see the prominent acetylated MARK2 protein band at ∼ 100 kDa). We note that the MARK2 expression plasmid used in the cell-based assays contains a myc tag and therefore migrates at ∼ 100 kD. CBP, CREB-binding protein; MARK2, microtubule affinity-regulating kinase 2.

We next considered the possibility that MARK2 and CBP engage in a feedback loop in which CBP may reciprocally modulate MARK2 activity to coordinate the phosphorylation and acetylation of their downstream substrates. Indeed, there is mounting evidence that kinases can be subjected to lysine acetylation leading to impaired catalytic activity (49, 50, 51, 52). Therefore, we examined the reciprocal process by which CBP would directly acetylate and inhibit MARK2 activity. First, we performed an *in vitro* acetylation reaction using recombinant purified proteins, in which the CBP catalytic domain was incubated with full-length GST-tagged MARK2 in the presence of acetyl-CoA (Fig. 3, *A* and *B*). CBP robustly acetylated recombinant MARK2, as determined by acetyl-lysine immunoreactivity (Fig. 3*A*, see acetyl-MARK2 band). As expected, CBP also undergoes prominent auto-acetylation in the presence of acetyl-CoA (53) (Fig. 3*A*, see band denoted by the asterisk).

To confirm the MARK2 acetylation findings in a cell-based model, we coexpressed CBP with each individual MARK2 variant, followed by immunoprecipitation/western blot analysis to determine MARK2 acetylation status. Similar to that shown in Figure 1, *A* and *D*, we observed the expected inhibition of CBP in the presence of active MARK2 (Fig. 3*C*, compare lane one to lanes 2–4). While all MARK2 variants were expressed, they showed different acetylation patterns in the presence of CBP (Fig. 3*D*). The partially active MARK-WT and constitutively active MARK2-TE showed negligible acetylation. In contrast, the inactive MARK2-KR was strongly acetylated (Fig. 3*D*, compare lanes 1–3 to lane 4), consistent with preferential CBP binding and acetylation of only the inactive MARK2 conformation.

**Figure 4:**
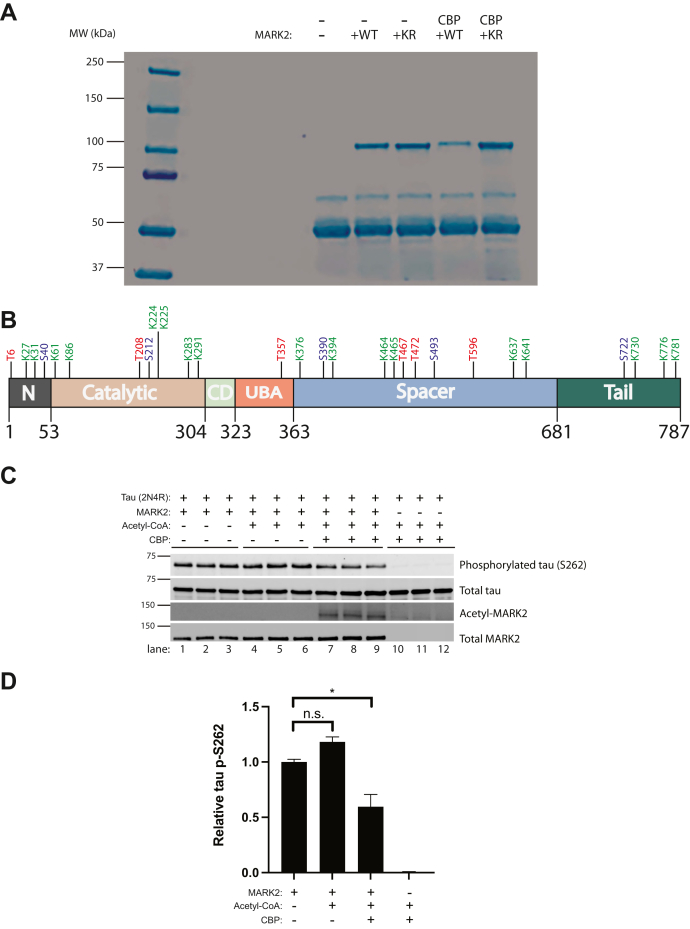
**PTM mapping identifies MARK2 as an acetylated substrate.***A*, 293A cells were transfected with a control vector (pcDNA5/TO) or MARK2 variants in the presence or absence of CBP-WT. Total cell lysates were extracted in low stringency NETN buffer, and myc-tagged MARK2 variants were immunopurified using myc antibodies conjugated to protein A/G beads. The Coomassie-stained gel shows the purified MARK2 protein at ∼ 100 kD that was subsequently gel-excised and analyzed by LC-MS/MS. *B*, a MARK2 molecular domain schematic illustrates the acetylated lysines (K) in *green*, phosphorylated serines (S) in *blue* and phosphorylated threonines (T) in *red*. In the presence of CBP-WT, a panel of MARK2 acetylation sites were identified and the peptide properties are described in Table S1. *C*, *in vitro* kinase reactions were performed in the presence or absence of MARK2-WT, CBP-WT, full-length tau-WT (2N4R), and acetyl-CoA, followed by immunoblotting analysis to determine whether MARK2 acetylation altered tau phosphorylation at residue S262. *D*, quantification of p-S262 across all conditions was determined by band densitometry. Error bars indicate SEM. n = 3 biologically independent experiments. *p* value was assessed by one-way ANOVA with Tukey’s post hoc test. n.s. *p* > 0.05; ∗*p* < 0.05. CBP, CREB-binding protein; MARK2, microtubule affinity-regulating kinase 2; PTM, posttranslational modification.

To determine the specific lysine residues in MARK2 that are subjected to acetylation, we immunopurified MARK2-KR alone or MARK2-KR that had been acetylated in the presence of CBP. We then confirmed the isolation by Coomassie detection of the ∼ 100 kD MARK2 protein band and analyzed gel-excised MARK2 by LC-MS/MS-based proteomics (Fig. 4*A*). We mapped 16 acetylated lysine residues spanning the entire MARK2 protein with the majority residing in the catalytic (K61, K86, K224/K225, K283, K291), spacer (K376, K394, K464, K465, K637, K641), and tail (K730, K776, K781) domains that regulate MARK2 kinase activity and/or protein–protein interactions (Fig. 4*B* and Table S1). All 16 acetylation sites were identified in the presence of CBP or were more abundant when CBP was present compared to MARK2-KR in the absence of CBP. We identified 11 phosphorylated residues, eight of which were less abundant in the presence of CBP (T6, S40, T208, S212, S390, T472, S493, S722), suggesting MARK2 acetylation may suppress its ability to undergo phosphorylation. All identified MARK2 acetylation and phosphorylation sites are described in Table S1.

Given the abundance of acetylated lysines within the catalytic domain combined with the preferential binding and acetylation of the inactive MARK2 variant, we suspected there might be a functional impact of acetylation on MARK2 kinase activity. We performed *in vitro* kinase assays in the presence of purified full-length (2N4R) tau, a well characterized MARK2 substrate (Fig. 4, *C* and *D*). In the presence of both CBP and acetyl-CoA, MARK2 became acetylated, and the acetylated MARK2 was associated with reduced tau phosphorylation at residue S262 within the MARK2-targeting KXGS motif (Fig. 4*C*, compare lanes 1–6 to lanes 7–9), an effect that corresponded to a ∼ 40% reduction in phosphorylated tau (Fig. 4*D*).

**Figure 5:**
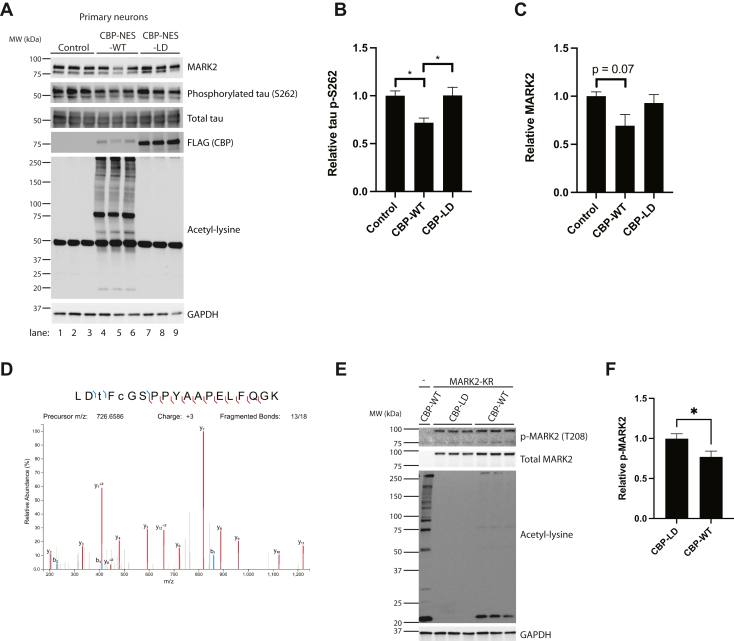
**CBP-mediated acetylation regulates MARK2 kinase activity.***A*–*C*, primary mouse cortical neurons were dissected, plated, and transduced at DIV3 with a lentivirus expressing either control (empty lentivirus), CBP-NES-WT (cytoplasmic CBP), or CBP-NES-LD (cytoplasmic inactive CBP). Neurons were harvested at DIV10 and analyzed by immunoblotting with MARK2, p-S262 tau, total tau, FLAG, and acetyl-lysine antibodies, while GAPDH served as a loading control. The extent of p-S262 and MARK2 were quantified and plotted in (*B*) and (*C*), which showed that tau phosphorylation at S262 is reduced in the presence of CBP-NES-WT. Error bars indicate SEM; n = 3 biologically independent experiments. *p* value was assessed by one-way ANOVA with Tukey’s post hoc test. ∗*p* < 0.05. *D*, gel-excised MARK2-WT was immunopurified from cell lysates, and global PTM site mapping was performed using LC-MS/MS. Purified MARK2 was reduced with DTT, alkylated with iodoacetamide, and digested in-gel with trypsin overnight, then subjected to LC-MS/MS analysis. MS/MS spectrum of the triply-charged ion at m/z 726.6586 corresponding to MARK2 peptide LDtFcGSPPYAAPELFQGK is shown. The spectrum provides evidence that residue T208 is in fact phosphorylated (an indicator of MARK2 activation status). *E* and *F*, 293A cells transfected with WT or catalytically inactive (LD) CBP variants in parallel with the kinase inactive variant of MARK2 (MARK2-KR) were immunoblotted with p-MARK2 (T208) or total MARK2 antibodies to analyze T208 phosphorylation status in response to acetylation. The ratio of P-MARK2 to total MARK2 (relative p-MARK2) was quantified and plotted in (f). Error bars indicate SEM; n = 6 biologically independent experiments. *p* value was assessed by unpaired Student’s *t* test. ∗*p* < 0.05. CBP, CREB-binding protein; MARK2, microtubule affinity-regulating kinase 2; NES, nuclear export signal; PTM, posttranslational modification.

To complement the recombinant *in vitro* MARK2 acetylation assays, we investigated whether MARK2 activity was similarly modulated by CBP in primary mouse cortical neurons. Lentiviruses were generated expressing only the active CBP catalytic domain fused to a nuclear export signal (CBP-NES-WT) to preferentially restrict CBP localization and acetyltransferase activity to the cytoplasm where MARK2 resides. This approach avoids any confounding issues and potential neurotoxicity resulting from the well-characterized nuclear effects of CBP on histone acetylation and gene transcription. CBP-NES-WT or the catalytically inactive control (designated as CBP-NES-LD) were transduced into cortical neurons at 3 days *in vitro* (DIV3) and tau phosphorylation at S262 was analyzed as a readout of MARK2 kinase activity. Despite the fact that CBP-NES-WT showed lower expression than that of CBP-NES-LD (which was more stable), CBP-NES-WT nonetheless showed robust cytoplasmic acetyltransferase activity, as shown by the increase in pan-acetyl-lysine immunoreactivity (Fig. 5*A*, lanes 4–6). In the presence of CBP-NES-WT, but not CBP-NES-LD, we observed a significant reduction in phosphorylated tau at S262, which coincided with a slight downward trend in total MARK2 levels, though the latter observation was not significant (Fig. 5, *A*–*C*). These findings support the notion that active cytoplasmic CBP can inhibit MARK2 kinase activity and thereby impact tau phosphorylation.

To provide further functional support for MARK2 acetylation impairing its kinase activity, we focused on a particular activating MARK2 phosphorylation event at the conserved T208 residue within the activation loop (38, 54). Our mass spectrometry data indicated that CBP led to a mild reduction in T208 phosphorylation, as indicated by a peptide abundance ratio < 1.0 (0.67) in the presence of CBP compared to the nonacetylated MARK2 control (Fig. 5*D* and Table S1, see phospho-MARK2 peptide spectrum). To confirm this observation, we performed immunoblotting analysis using a phospho-MARK2 specific antibody detecting phosphorylated residue T208, the quantification of which again showed a modest reduction in the overall ratio of phosphorylated MARK2 relative to the total MARK2 pool (Fig. 5, *E* and *F*), confirming altered MARK2 activity upon acetylation by CBP.

### MARK2 protein levels are reduced in tauopathy brain

**Figure 6:**
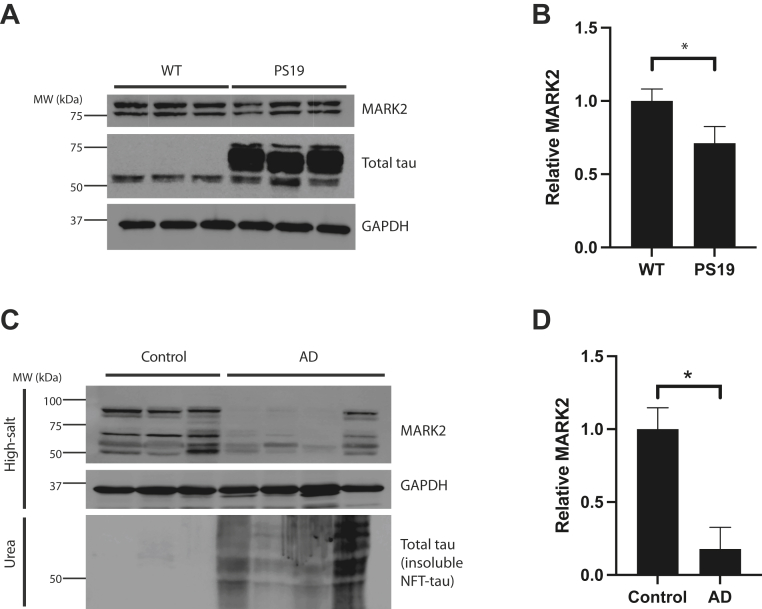
**MARK2 protein levels are reduced in tauopathy mice and human AD.***A* and *B*, the cortex was isolated from 12-month-old WT and PS19 mice and extracted using high-salt buffer. High-salt fractions were analyzed by immunoblotting using MARK2 or total tau (TAU-5) antibodies. The relative levels of MARK2 protein were quantified in (*B*). Error bars indicate SEM. n = 3 biologically independent experiments. *p* value was assessed by unpaired Student’s *t* test. ∗*p* < 0.05. *C* and *D*, high-salt soluble and urea-extracted insoluble fractions of cortical brain homogenates from control or AD brain tissue was analyzed by immunoblotting using MARK2 or total tau (TAU-5) antibodies. The urea-extracted tau is present only in AD brain, confirming the presence of tau pathology. GAPDH served as a loading control. The levels of MARK2 in the high-salt fraction were quantified in (*D*). The molecular weight of MARK2 is ∼ 77 to 90 kD, corresponding to the protein doublets observed in mouse and human tissues. For full data transparency, all protein bands are displayed including possible MARK2 breakdown products < 75 kDa. Error bars indicate SEM. n = 3 biologically independent experiments. *p* value was assessed by unpaired Student’s *t* test. ∗*p* < 0.05. AD, Alzheimer’s disease; MARK2, microtubule affinity-regulating kinase 2.

These data support a reciprocal regulatory pathway in which MARK2 modulates CBP, and conversely that CBP inhibits MARK2 activity *via* direct acetylation. Recent studies suggest that abnormal CBP activity and excessive acetylation of downstream substrates (including but not limited to tau) occurs in human AD brain (7, 8) and can accelerate general protein aggregation and the formation of toxic amyloids (46). Therefore, we asked whether reduced MARK2 protein levels (and hence increased CBP activity) is a feature of AD brain. We first analyzed MARK2 levels in cortical brain samples from 12-month-old WT and symptomatic tau P301S (PS19) transgenic mice that display tau pathology, neuronal loss, and cognitive impairments (55). Using a total MARK2-specific antibody, we found a modest yet significant reduction in MARK2 protein levels in PS19 mice coincident with tau accumulation (Fig. 6, *A* and *B*). We next analyzed MARK2 levels in fractionated human control and AD postmortem brains. We confirmed the extensive NFT pathology in AD brain by evaluating tau aggregation in insoluble fractions, which showed the expected pattern of insoluble aggregated and smeared tau that is present only in AD brain samples (Fig. 6*C*). Similar to tauopathy mice, we found significantly lower MARK2 protein levels in AD brains, again consistent with a negative correlation between MARK2 levels and AD progression (Fig. 6, *C* and *D*). Overall, these findings suggest that MARK2 dysfunction and altered downstream regulation of MARK2 substrates is associated with the progression of tauopathy.

## Discussion

In this study, we demonstrate that MARK2 operates as part of a negative feedback loop with the acetyltransferase CBP, potentially to coordinate downstream acetylation and phosphorylation events. We provide evidence that MARK2 reduces the levels (and therefore activity of CBP) and decreases lysine acetylation globally, as detected with pan-acetylation antibodies. We did not observe direct MARK2-mediated phosphorylation of CBP, but rather we found that MARK2 may facilitate the destabilization or degradation of CBP, as CBP levels were reduced in the presence of active MARK2 (Fig. 2*C*) and increased in the presence of a MARK2 pharmacological inhibitor (Fig. 2, *D* and *E*). Consistent with reduction or inhibition of MARK2 in AD (Fig. 6, *C* and *D*), increased CBP/p300 activity and histone H3K27/H3K9 acetylation were observed in AD brain (7, 8). Additional effort beyond this study is needed to understand mechanistically how MARK2 could deplete CBP levels. For example, a prior study implicated a caspase-6-dependent mechanism that results in CBP cleavage (56), and therefore MARK2 could potentially facilitate caspase-dependent CBP cleavage. In addition, a recent study demonstrated that MARK2 can influence protein translation *via* phosphorylation of eIF2α, which could also regulate CBP expression (57).

Our findings show that inactive MARK2 (*i.e.*, MARK2-KR) is preferentially bound (Fig. 2*B*) and acetylated by CBP (Fig. 3*D*). This effect is likely to be direct since CBP was able to acetylate MARK2 using purified recombinant proteins in the presence of acetyl-CoA (Fig. 3, *A* and *B*). Mass spectrometry-based PTM mapping indicated a significant number of acetylated residues that reside in the catalytic and spacer domains, implying that acetylation negatively regulates MARK2 kinase activity (Fig. 4*B* and Table S1). Outside the catalytic domain, another acetylation site of interest (K61) resides in the P-loop (residues 60–65) (Fig. 4*B*), which facilitates conversion to the active state (36, 58). Using kinase activity assays in which tau was included as a MARK2 substrate, we found that acetylated MARK2 was associated with impaired kinase activity directed toward the KXGS motif within tau’s MTBR domain (S262) (Fig. 4, *C* and *D*). While tau is considered a well-studied and easily detected MARK2 substrate, future studies will be needed to evaluate other MARK2 substrates beyond tau including related MAPs (59), kinesin family proteins (60, 61), and stress response factors (*e.g.*, eIF2α) (57).

We suspect that physical binding of CBP to MARK2 followed by MARK2 acetylation are likely required to maintain MARK2 in the inactive state. Several identified MARK2 acetylation sites were clustered in the catalytic domain, including residue(s) K224/K225 in close proximity to the activating phosphorylation site at residue T208, suggesting MARK2 acetylation could prevent nearby Ser/Thr phosphorylation (Fig. 4*B* and Table S1). There is emerging evidence for lysine acetylation as a dominant regulatory mechanism to control kinase activity, as acetylation targets the MAPK family of kinases (62, 63), SIK2 (64), p70 ribosomal S6 kinase (65), the major AMPK kinase LKB1 (66), and cyclin-dependent kinases cdk2 (51) and cdk9 (52, 67). The growing list of kinase targets suggests that acetylation–phosphorylation interplay may be more broadly applicable to kinase biology, acting as molecular switch to control signaling pathways largely driven by kinase activity.

Based on our initial findings, we expected an inverse relationship between MARK2 and tauopathy progression, though no prior studies had documented MARK2 protein changes in AD brain. Currently, we can only correlate MARK2 levels with AD and cannot assert any causative link between reduced MARK2 levels and tau pathology. For example, it is equally possible that AD progression leads to a reduction in MARK2 levels rather than reduced MARK2 levels driving tau pathology. However, if the latter scenario is correct, reduced MARK2 levels could result in elevated or activated CBP that is capable of generating the abnormally acetylated tau species that we and others have documented in AD and other tauopathies. Indeed, we found a five-fold reduction in MARK2 protein levels in AD compared to controls (Fig. 6*D*). While the scenario in which MARK2 levels are depleted is consistent with the activation of CBP, it is not consistent with the accumulation of phosphorylated tau at S262 that is so prominent in AD brain and associated with NFTs (68). However, it is important to note that other tau kinases including related MARK family members (69, 70), GSK-3 (71), and PKA (72) are thought to target the same S262 residue within the KXGS motifs. Therefore, MARK2 protein levels are not necessarily a proxy for S262 phosphorylated tau. Whether the levels of other tau kinases change in parallel with MARK2 in tauopathy models will require further investigation.

While aberrant PTMs including phosphorylation and acetylation are clearly implicated in aging and neurodegeneration, their functional interplay is unclear. Our study provides new evidence for a negative regulatory mechanism between a kinase (MARK2) and an acetyltransferase (CBP), two master PTM–remodeling enzymes. These findings could lay the foundation to explore distinct PTM codes in the brain and perhaps guide future therapeutic efforts to target disease pathology by modulating PTM feedback loops.

## Experimental procedures

### Plasmids

The following expression plasmids were used in this study and, where indicated, mutations were generated by site-directed mutagenesis. All CBP or MARK2 expression plasmids for transient transfections were cloned into the pcDNA5/TO vector (Life Technologies). FLAG-tagged WT full-length CBP (CBP-WT) and inactive CBP containing L1435A/D1436A mutations (CBP-LD) were used to promote acetylation. Myc-tagged WT full-length MARK2 (MARK2-WT), constitutively active MARK2-T208E (MARK2-TE), and inactive MARK2-K82R (MARK2-KR) were used to monitor kinase activity. The detailed descriptions of the plasmids used in this study can be found in Table S2.

### Cell culture

293A cells (Invitrogen) are commercially available and were grown in full Dulbecco’s Modified Eagle Media (DMEM, Gibco) supplemented with 10% fetal bovine serum (Sigma), 1X L-glutamine (Gibco), and 1X penicillin/streptomycin (Sigma). This cell line is a subclone of the standard HEK293 line with a relatively flat morphology, is more slowly growing, and maintains ectopic plasmid expression at more physiological levels rather than supra-physiological overexpression. Plasmids were transfected into 293A cells using Fugene 6 Transfection Reagent (Promega) and incubated for 48 h prior to harvest to ensure robust expression and sensitive detection.

### Lentivirus cloning and generation

To generate lentiviral expression plasmids for CBP-NES-WT and CBP-NES-LD, the mammalian expression vectors for flag-CBP-NES-WT and flag-CBP-NES-LD (cloned into pcDNA3.1) were used as templates and amplified by PCR (with a FLAG-tag at the 5′ end). The fragments were inserted into the pUltra vector using AgeI and SalI restriction endonucleases to replace the eGFP cassette. A control (empty) lentiviral vector (pUltraXeGFP) was generated by removing the eGFP cassette by digestion with AgeI and BsrGI, and ligation was performed after Klenow blunting. Lentiviral production was performed by cotransfecting 37.5 μg lenti-plasmid with 25 μg psPAX2, 12.5 μg VSVG, and 6.25 μg REV for each 15 cm dish of lenti-X 293T cells (CalPhos Transfection Kit, Takara). Three 15 cm dishes of cells were used for each lentiviral production. Three days after transfection, culture media were collected and centrifuged at 2000*g* for 10 min. Lentiviral particles were purified using a double-sucrose gradient method. Briefly, the supernatants were loaded onto a 70%-60%-30%-20% sucrose gradient and centrifuged at 70,000*g* for 2 h at 17 °C (using a Beckman Optima LE-80K ultracentrifuge). The 30%-60% fraction containing the viral particles was retrieved, resuspended in PBS, filtered with a 0.45 μm filter flask before loaded onto a 20% sucrose cushion, and centrifuged a second time at 70,000*g* for 2 h at 17 °C. The supernatants were carefully discarded and the viral particles present in the pellet were resuspended in PBS, aliquoted, and stored at −80 °C. The details of the specific lentiviral constructs used in this study can be found in Table S2.

### Biochemical assays and immunoblotting

Biochemical analyses for preparation of lysates were performed as follows using a sequential extraction technique to generate soluble and insoluble lysates. Before harvest, cells (either 293A cells or primary mouse cortical neurons) were washed with PBS. Cells were harvested in RIPA buffer (50 mM Tris pH 8.0, 0.15 M NaCl, 5 mM EDTA, 12.06 mM sodium deoxycholate, 1% v/v IGEPAL CA-630, 0.1% w/v SDS) supplemented with deacetylase (100 mM trichostatin A and 10 mM nicotinamide; Sigma), phosphatase (1 mM β-glycerophosphate, 1 mM NaF, 1 mM sodium orthovanadate; Sigma) and protease inhibitors (1 mg/ml pepstatin, leupeptin, N-p-tosyl-L-phenylalanine chloromethyl ketone, Na-Tosyl-L-lysine chloromethyl ketone hydrochloride, trypsin inhibitor; Sigma). Cells were sonicated 20 times and centrifuged at 21,130*g* for 30 min at 4 °C. The supernatant was collected as the RIPA (soluble) fraction. The resulting pellet was washed in RIPA buffer and then re-extracted in Urea buffer (7 M urea, 2 M thiourea, 4% CHAPS, 30 mM Tris pH 8.5) supplemented with deacetylase, phosphatase, and protease inhibitors (as described above). The samples were sonicated 10 times and centrifuged at 21,130*g* for 30 min at room temperature. The supernatant was collected as RIPA-insoluble (insoluble) fraction. The samples were then analyzed using SDS-PAGE, transferred to nitrocellulose membrane (Biorad), and blocked with 2% milk in 1X TBS for 30 min. Membranes were incubated with primary antibodies, as indicated, overnight at 4 °C, followed by a 1 h room temperature incubation with the secondary antibodies conjugated to horseradish peroxidase (1:1000, Thermo #31430 or #32460). The following primary antibodies were used in this study: acetyl-lysine (1:1000, Cell Signaling #9441), Myc (1:1000, Santa Cruz #sc-40), MARK2 (1:1000, Cell Signaling #9118), p-MARK2 (1:1000, Cell Signaling #4836), FLAG (1:1000, Sigma Aldrich #F1804), tau p-S262 (1:1000, Invitrogen #44-750G), total tau K9JA (1:5000, DAKO #A0024), total tau TAU-5 (1:1000, Invitrogen #AHB0042), CBP (1:1000, Cell Signaling #7389), and GAPDH (1:1000, Millipore #ABS16). An anti-phosphorylated Ser/Thr/Tyr antibody cocktail was used to detect phosphorylated epitopes on immunoprecipitated CBP (1:4000, Cell Signaling #5759, #9477, #6967, #9614, and #6966),. Membranes were developed with ECL Western Blotting Substrate (ThermoFisher) and imaged using Image Quant LAS 4000 (Cytiva). Image Quant TL (Cytiva) or Image Studio Lite (LI-COR Biosciences) was used to quantify and plot protein band intensities.

### Coimmunoprecipitation assay

For co-IP studies, 293A cells transfected with desired plasmids were lysed in a low stringency NETN buffer suitable for interaction analysis (50 mM Tris–HCl, pH 7.4, 150 mM NaCl, 1 mM EDTA, 1% NP-40, supplemented with deacetylase, phosphatase, and protease inhibitors as described above) to maintain protein–protein interactions. Soluble supernatants (1 mg total protein) were cleared by centrifugation at 21,130*g* for 30 min, incubated overnight with protein A/G beads (Santa Cruz) complexed to the indicated antibodies (myc or FLAG), and subsequently analyzed by SDS-PAGE and immunoblotting. Analyses and quantification of co-IP assays were as described in the Biochemical assays and immunoblotting and Statistical analysis sections.

### LC-MS/MS analysis

The inactive MARK2-KR variant (which is bound and acetylated by CBP) was transfected in the absence or presence of CBP-WT into 293A cells. Immunoprecipitation followed by mass spectrometry (LC-MS/MS) was performed. Samples were run on a 4 to 15% Criterion TGX Stain-Free Protein Gel and stained according to the SimplyBlue SafeStain protocol (Invitrogen). The protein bands were submitted to the UNC Proteomics Core for LC-MS/MS analysis in order to map PTM sites on MARK2. Briefly, gel bands corresponding to MARK2 were excised, and the proteins were reduced, alkylated, and in-gel digested with trypsin overnight at 37 °C. Peptides were extracted, desalted with C18 spin columns (Pierce), and dried *via* vacuum centrifugation. Peptide samples were stored at −80 °C until further analysis. The peptide samples (n = 2) were analyzed by LC/MS/MS using an Easy nLC 1200 coupled to a QExactive HF mass spectrometer (Thermo Scientific). Samples were injected onto an Easy Spray PepMap C18 column (75 μm id × 25 cm, 2 μm particle size) (Thermo Scientific) and separated over a 60 min method. The gradient for separation consisted of 5 to 40% mobile phase B at a 250 nl/min flow rate, where mobile phase A was 0.1% formic acid in water and mobile phase B consisted of 0.1% formic acid in 80% ACN. The QExactive HF was operated in data-dependent mode where the 15 most intense precursors were selected for subsequent fragmentation. Resolution for the precursor scan (m/z 350–1600) was set to 120,000 with a target value of 3 × 10^6^ ions. MS/MS scans resolution was set to 15,000 with a target value of 1 × 10^5^ ions. The normalized collision energy was set to 27% for HCD. Dynamic exclusion was set to 30 s, peptide match was set to preferred, and precursors with unknown charge or a charge state of 1 and ≥ 8 were excluded.

### LC-MS/MS data analysis

Raw data files were processed using Proteome Discoverer version 2.5 (Thermo Scientific). Peak lists were searched against a reviewed Uniprot human database (containing 20,350 protein sequences, downloaded in Feb 2020), appended with a common contaminants database, using Sequest. The following parameters were used to identify tryptic peptides for protein identification: 20 ppm precursor ion mass tolerance; 0.02 Da product ion mass tolerance; up to three missed trypsin cleavage sites; (C) carbamidomethylation was set as a fixed modification; (M) oxidation, (S, T, Y) phosphorylation, and (K, N-terminus) acetylation were set as variable modifications. The ptmRS node was used to localize the sites of phosphorylation and acetylation. Peptide false discovery rates were calculated by the Percolator node using a decoy database search and data were filtered using a 1% false discovery rate cutoff. The Minora node was used to extract peak areas for relative quantitation of the PTM peptides. Peak area ratios were calculated by dividing the peak area of the MARK2-KR + CBP-WT sample by the peak area of MARK2-KR alone control for the PTM peptides. PTM peptides were manually validated. MS/MS spectrum of the phosphorylated T208 peptide was annotated using IPSA (https://pubmed.ncbi.nlm.nih.gov/31088857/). The mass spectrometry proteomics data have been deposited to the PRIDE Archive (http://www.ebi.ac.uk/pride/archive/) *via* the PRIDE partner repository with the data set identifier PXD030937.

### *In vitro* acetylation assay

To determine whether MARK2 could undergo acetylation *in vitro*, reactions containing a combination of 400 ng recombinant GST-tagged MARK2 (Sigma #SRP5045), 1 μl CBP catalytic domain (Millipore #03189), or 1 μM acetyl-CoA (Sigma #A2056) were mixed in acetylation buffer (50 mM Tris–HCl pH 8.0, 10% glycerol, 1 mM DTT, 100 mM EDTA) to initiate the *in vitro* acetylation and incubated for 1.5 h at 37 °C. The reaction was terminated by adding 6X SDS sample loading buffer, and the ability of CBP to acetylate MARK2 was determined by pan-acetyl-lysine immunoblotting (Cell Signaling, #9441)

### *In vitro* kinase assay

To determine whether MARK2 acetylation impacted MARK2 kinase function, we performed standard *in vitro* kinase assays. Reactions containing a combination of 4 μg recombinant full-length tau (2N4R), 1 μg GST-tagged MARK2 (Sigma #SRP5045), 2 μl CBP catalytic domain (Millipore #03189), or 1 μM acetyl-CoA (Sigma #A2056) were mixed in the kinase buffer (50 mM Tris–HCl pH 7.2, 10% glycerol, 1 mM DTT, 100 μM EDTA) to initiate *in vitro* kinase reaction for 1 h at 30 °C. The reaction was terminated by adding 6X SDS sample loading buffer, and the kinase activity of MARK2 on tau substrate was determined by the extent of tau phosphorylation at a preferred MARK2 target site on tau (phosphorylated Ser-262).

### Primary mouse neurons and lentiviral transduction

Primary neuron cultures were prepared from embryonic day (E) 15 to 16 embryos of nontransgenic C57BL/6 mice (Charles River). All procedures were performed in strict compliance with animal protocols approved by the Institutional Animal Care and Use Committee of the University of North Carolina at Chapel Hill (#21.257). The mice were lethally anesthetized in isoflurane and the uterus was removed and placed in cold Hepes-buffered Hank’s balanced salt solution. The fetuses were removed and the brain was harvested from the cranium. The cerebral hemispheres were minced and digested in the presence of 20 U/ml papain (Worthington) and 5U/ml DNase (Promega) for 30 min at 37 °C. Tissue was dissociated mechanically using a P1000 pipette. The cell suspension was passed through a 40 μm cell strainer (Corning, #352340). Dissociated neurons were counted and plated onto poly-D-lysine (Sigma)–coated coverslips or plates. Lentiviruses (control, CBP-NES-WT, or CBP-NES-LD) were transduced into primary neurons at DIV3, incubated for 7 days, and cells were harvested at DIV10 for further analysis. Where indicated, 20 μM MARK2 inhibitor 39621 (Millipore, #454870) was used treated to primary neurons at DIV14 for 5 h to suppress MARK2 kinase activity.

### Mouse and human brain homogenate preparation

WT and PS19 mice at 12 months old were sacrificed, and isolated cortex was homogenized in 4 vol/g of high-salt buffer (10 mM Tris base, 500 mM NaCl and 2 mM EDTA) supplemented with deacetylase, phosphatase, and protease inhibitors as described above and centrifuged at 21,130*g* for 45 min to generate high-salt fractions. The high-salt soluble fractions were then analyzed by SDS-PAGE and immunoblotting using the indicated antibodies to detect MARK2 and total tau proteins. Human control and AD brain tissues from Braak V-VI cases were kindly provided by the University of Pennsylvania (Center for Neurodegenerative Disease Research brain bank). Isolated gray matter from frontal cortex was homogenized in 3 vol/g of cold high-salt RAB buffer (0.75 M NaCl, 100 mM Tris, 1 mM EGTA, 0.5 mM MgSO4, 0.02 M NaF, 2 mM DTT, pH 7.4) supplemented with deacetylase, phosphatase, and protease inhibitors as described above. Homogenates were incubated at 4 °C for 20 min to depolymerize MTs, then, centrifuged at 100,000*g* for 30 min at 4 °C. The resulting supernatant is labeled as the high-salt fraction. Pellets were re-homogenized and centrifuged in 3 vol/g of cold high-salt RAB buffer. Resultant pellets were homogenized in 5 vol/g of cold RIPA buffer (50 mM Tris pH 8.0, 150 mM NaCl, 1% NP-40, 5 mM EDTA, 0.5% sodium deoxycholate, 0.1% SDS) and centrifuged at 100,000*g* for 30 min at 4 °C. Myelin floatation was performed on pellets re-extracted in RIPA buffer supplemented with 20% sucrose to remove the excess myelin present in human brain homogenates. Finally, resultant insoluble pellets were extracted in 1 vol/g urea extraction buffer (7 M urea, 2 M Thiourea, 4% CHAPS, 30 mM Tris, pH 8.5). High-salt and urea fractions were analyzed by SDS-PAGE and immunoblotting using the indicated antibodies to detect MARK2 and total tau proteins.

### Statistical analyses

GraphPad Prism 9 software was used for all statistical analyses. Results were pooled from a minimum of three independent experiments and presented as average ± SEM. Comparisons between two groups were analyzed using unpaired Student’s *t* test (where total groups = 2) or one-way ANOVA with Tukey’s post hoc test (where total groups ≥ 3). Significance is presented as n.s. *p* > 0.05, ∗*p* < 0.05, or ∗∗∗∗*p* < 0.0001.

## Data availability

Mass spectrometry data was deposited in the PRIDE Archive with the data set identifier PXD030937. All other data are contained within the article and supporting information.

## Supporting information

This article contains supporting information.

## Conflict of interest

The authors declare that they have no conflicts of interest with the contents of this article.

## Supporting information

Table S1.xlsx; APPLICATION

Table S2.docx; APPLICATION

Figure S1.pdf; IMAGE

## References

[1] Santos A.L., Lindner A.B. (2017). Protein posttranslational modifications: Roles in aging and age-related disease. Oxid Med. Cell Longev..

[2] Shafi S., Singh A., Gupta P., Chawla P.A., Fayaz F., Sharma A., Pottoo F.H. (2021). Deciphering the role of aberrant protein post-translational modification in the pathology of neurodegeneration. CNS Neurol. Disord. Drug Targets.

[3] Taylor B.C., Young N.L. (2021). Combinations of histone post-translational modifications. Biochem. J..

[4] Wen J., Wang D. (2022). Deciphering the PTM codes of the tumor suppressor p53. J. Mol. Cell Biol..

[5] Wu Z., Huang R., Yuan L. (2019). Crosstalk of intracellular post-translational modifications in cancer. Arch. Biochem. Biophys..

[6] Ramazi S., Allahverdi A., Zahiri J. (2020). Evaluation of post-translational modifications in histone proteins: A review on histone modification defects in developmental and neurological disorders. J. Biosci..

[7] Nativio R., Donahue G., Berson A., Lan Y., Amlie-Wolf A., Tuzer F., Toledo J.B., Gosai S.J., Gregory B.D., Torres C., Trojanowski J.Q., Wang L.S., Johnson F.B., Bonini N.M., Berger S.L. (2018). Dysregulation of the epigenetic landscape of normal aging in Alzheimer's disease. Nat. Neurosci..

[8] Nativio R., Lan Y., Donahue G., Sidoli S., Berson A., Srinivasan A.R., Shcherbakova O., Amlie-Wolf A., Nie J., Cui X., He C., Wang L.S., Garcia B.A., Trojanowski J.Q., Bonini N.M. (2020). An integrated multi-omics approach identifies epigenetic alterations associated with Alzheimer's disease. Nat. Genet..

[9] Alquezar C., Arya S., Kao A.W. (2020). Tau post-translational modifications: Dynamic transformers of tau function, degradation, and aggregation. Front. Neurol..

[10] Saha P., Sen N. (2019). Tauopathy: A common mechanism for neurodegeneration and brain aging. Mech. Ageing Dev..

[11] Manzanza N.O., Sedlackova L., Kalaria R.N. (2021). Alpha-synuclein post-translational modifications: Implications for pathogenesis of lewy body disorders. Front. Aging Neurosci..

[12] Lontay B., Kiss A., Virag L., Tar K. (2020). How do post-translational modifications influence the pathomechanistic landscape of huntington's disease? A comprehensive review. Int. J. Mol. Sci..

[13] Schaffert L.N., Carter W.G. (2020). Do post-translational modifications influence protein aggregation in neurodegenerative diseases: A systematic review. Brain Sci..

[14] Farina S., Esposito F., Battistoni M., Biamonti G., Francia S. (2021). Post-translational modifications modulate proteinopathies of TDP-43, FUS and hnRNP-A/B in amyotrophic lateral sclerosis. Front. Mol. Biosci..

[15] Sternburg E.L., Gruijs da Silva L.A., Dormann D. (2021). Post-translational modifications on RNA-binding proteins: Accelerators, brakes, or passengers in neurodegeneration?. Trends Biochem. Sci..

[16] Cho J.H., Johnson G.V. (2003). Glycogen synthase kinase 3beta phosphorylates tau at both primed and unprimed sites. Differential impact on microtubule binding. J. Biol. Chem..

[17] Cho J.H., Johnson G.V. (2004). Primed phosphorylation of tau at Thr231 by glycogen synthase kinase 3beta (GSK3beta) plays a critical role in regulating tau's ability to bind and stabilize microtubules. J. Neurochem..

[18] Liu S.J., Zhang J.Y., Li H.L., Fang Z.Y., Wang Q., Deng H.M., Gong C.X., Grundke-Iqbal I., Iqbal K., Wang J.Z. (2004). Tau becomes a more favorable substrate for GSK-3 when it is prephosphorylated by PKA in rat brain. J. Biol. Chem..

[19] Steinhilb M.L., Dias-Santagata D., Fulga T.A., Felch D.L., Feany M.B. (2007). Tau phosphorylation sites work in concert to promote neurotoxicity *in vivo*. Mol. Biol. Cell.

[20] Steinhilb M.L., Dias-Santagata D., Mulkearns E.E., Shulman J.M., Biernat J., Mandelkow E.M., Feany M.B. (2007). S/P and T/P phosphorylation is critical for tau neurotoxicity in Drosophila. J. Neurosci. Res..

[21] Arakhamia T., Lee C.E., Carlomagno Y., Duong D.M., Kundinger S.R., Wang K., Williams D., DeTure M., Dickson D.W., Cook C.N., Seyfried N.T., Petrucelli L., Fitzpatrick A.W.P. (2020). Posttranslational modifications mediate the structural diversity of tauopathy strains. Cell.

[22] Carlomagno Y., Chung D.C., Yue M., Castanedes-Casey M., Madden B.J., Dunmore J., Tong J., DeTure M., Dickson D.W., Petrucelli L., Cook C. (2017). An acetylation-phosphorylation switch that regulates tau aggregation propensity and function. J. Biol. Chem..

[23] Cook C., Carlomagno Y., Gendron T.F., Dunmore J., Scheffel K., Stetler C., Davis M., Dickson D., Jarpe M., Deture M., Petrucelli L. (2014). Acetylation of the KXGS motifs in tau is a critical determinant in modulation of tau aggregation and clearance. Hum. Mol. Genet..

[24] Cohen T.J., Guo J.L., Hurtado D.E., Kwong L.K., Mills I.P., Trojanowski J.Q., Lee V.M. (2011). The acetylation of tau inhibits its function and promotes pathological tau aggregation. Nat. Commun..

[25] Min S.W., Cho S.H., Zhou Y., Schroeder S., Haroutunian V., Seeley W.W., Huang E.J., Shen Y., Masliah E., Mukherjee C., Meyers D., Cole P.A., Ott M., Gan L. (2010). Acetylation of tau inhibits its degradation and contributes to tauopathy. Neuron.

[26] Dorval V., Fraser P.E. (2006). Small ubiquitin-like modifier (SUMO) modification of natively unfolded proteins tau and alpha-synuclein. J. Biol. Chem..

[27] Luo H.B., Xia Y.Y., Shu X.J., Liu Z.C., Feng Y., Liu X.H., Yu G., Yin G., Xiong Y.S., Zeng K., Jiang J., Ye K., Wang X.C., Wang J.Z. (2014). SUMOylation at K340 inhibits tau degradation through deregulating its phosphorylation and ubiquitination. Proc. Natl. Acad. Sci. U. S. A..

[28] Funk K.E., Thomas S.N., Schafer K.N., Cooper G.L., Liao Z., Clark D.J., Yang A.J., Kuret J. (2014). Lysine methylation is an endogenous post-translational modification of tau protein in human brain and a modulator of aggregation propensity. Biochem. J..

[29] Huseby C.J., Hoffman C.N., Cooper G.L., Cocuron J.C., Alonso A.P., Thomas S.N., Yang A.J., Kuret J. (2019). Quantification of tau protein lysine methylation in aging and Alzheimer's disease. J. Alzheimer's Dis..

[30] Thomas S.N., Funk K.E., Wan Y., Liao Z., Davies P., Kuret J., Yang A.J. (2012). Dual modification of Alzheimer's disease PHF-tau protein by lysine methylation and ubiquitylation: A mass spectrometry approach. Acta Neuropathol..

[31] Cripps D., Thomas S.N., Jeng Y., Yang F., Davies P., Yang A.J. (2006). Alzheimer disease-specific conformation of hyperphosphorylated paired helical filament-Tau is polyubiquitinated through Lys-48, Lys-11, and Lys-6 ubiquitin conjugation. J. Biol. Chem..

[32] Morishima-Kawashima M., Hasegawa M., Takio K., Suzuki M., Titani K., Ihara Y. (1993). Ubiquitin is conjugated with amino-terminally processed tau in paired helical filaments. Neuron.

[33] Alam J., Sharma L. (2019). Potential enzymatic targets in Alzheimer's: A comprehensive review. Curr. Drug Targets.

[34] Annadurai N., Agrawal K., Dzubak P., Hajduch M., Das V. (2017). Microtubule affinity-regulating kinases are potential druggable targets for Alzheimer's disease. Cell Mol. Life Sci..

[35] Turab Naqvi A.A., Hasan G.M., Hassan M.I. (2020). Targeting tau hyperphosphorylation *via* kinase inhibition: Strategy to address Alzheimer's disease. Curr. Top Med. Chem..

[36] Panneerselvam S., Marx A., Mandelkow E.M., Mandelkow E. (2006). Structure of the catalytic and ubiquitin-associated domains of the protein kinase MARK/Par-1. Structure.

[37] Gu G.J., Wu D., Lund H., Sunnemark D., Kvist A.J., Milner R., Eckersley S., Nilsson L.N., Agerman K., Landegren U., Kamali-Moghaddam M. (2013). Elevated MARK2-dependent phosphorylation of Tau in Alzheimer's disease. J. Alzheimer's Dis..

[38] Timm T., Li X.Y., Biernat J., Jiao J., Mandelkow E., Vandekerckhove J., Mandelkow E.M. (2003). MARKK, a Ste20-like kinase, activates the polarity-inducing kinase MARK/PAR-1. EMBO J..

[39] Chen X., Li Y., Wang C., Tang Y., Mok S.A., Tsai R.M., Rojas J.C., Karydas A., Miller B.L., Boxer A.L., Gestwicki J.E., Arkin M., Cuervo A.M., Gan L. (2020). Promoting tau secretion and propagation by hyperactive p300/CBP *via* autophagy-lysosomal pathway in tauopathy. Mol. Neurodegener..

[40] Hwang A.W., Trzeciakiewicz H., Friedmann D., Yuan C.X., Marmorstein R., Lee V.M., Cohen T.J. (2016). Conserved lysine acetylation within the microtubule-binding domain regulates MAP2/tau family members. PLoS One.

[41] Kamah A., Huvent I., Cantrelle F.X., Qi H., Lippens G., Landrieu I., Smet-Nocca C. (2014). Nuclear magnetic resonance analysis of the acetylation pattern of the neuronal Tau protein. Biochemistry.

[42] Portillo M., Eremenko E., Kaluski S., Garcia-Venzor A., Onn L., Stein D., Slobodnik Z., Zaretsky A., Ueberham U., Einav M., Bruckner M.K., Arendt T., Toiber D. (2021). SIRT6-CBP-dependent nuclear Tau accumulation and its role in protein synthesis. Cell Rep..

[43] Morris M., Knudsen G.M., Maeda S., Trinidad J.C., Ioanoviciu A., Burlingame A.L., Mucke L. (2015). Tau post-translational modifications in wild-type and human amyloid precursor protein transgenic mice. Nat. Neurosci..

[44] Wesseling H., Mair W., Kumar M., Schlaffner C.N., Tang S., Beerepoot P., Fatou B., Guise A.J., Cheng L., Takeda S., Muntel J., Rotunno M.S., Dujardin S., Davies P., Kosik K.S. (2020). Tau PTM profiles identify patient heterogeneity and stages of Alzheimer's disease. Cell.

[45] Ahrari S., Mogharrab N. (2014). Effects of T208E activating mutation on MARK2 protein structure and dynamics: Modeling and simulation. Mol. Biol. Res. Commun..

[46] Olzscha H., Fedorov O., Kessler B.M., Knapp S., La Thangue N.B. (2017). CBP/p300 bromodomains regulate amyloid-like protein aggregation upon aberrant lysine acetylation. Cell Chem. Biol..

[47] Huang W.C., Ju T.K., Hung M.C., Chen C.C. (2007). Phosphorylation of CBP by IKKalpha promotes cell growth by switching the binding preference of CBP from p53 to NF-kappaB. Mol. Cell.

[48] Impey S., Fong A.L., Wang Y., Cardinaux J.R., Fass D.M., Obrietan K., Wayman G.A., Storm D.R., Soderling T.R., Goodman R.H. (2002). Phosphorylation of CBP mediates transcriptional activation by neural activity and CaM kinase IV. Neuron.

[49] Lee J., Ko Y.U., Chung Y., Yun N., Kim M., Kim K., Oh Y.J. (2018). The acetylation of cyclin-dependent kinase 5 at lysine 33 regulates kinase activity and neurite length in hippocampal neurons. Sci. Rep..

[50] Lee J., Yun N., Kim C., Song M.Y., Park K.S., Oh Y.J. (2014). Acetylation of cyclin-dependent kinase 5 is mediated by GCN5. Biochem. Biophys. Res. Commun..

[51] Mateo F., Vidal-Laliena M., Canela N., Zecchin A., Martinez-Balbas M., Agell N., Giacca M., Pujol M.J., Bachs O. (2009). The transcriptional co-activator PCAF regulates cdk2 activity. Nucl. Acids Res..

[52] Sabo A., Lusic M., Cereseto A., Giacca M. (2008). Acetylation of conserved lysines in the catalytic core of cyclin-dependent kinase 9 inhibits kinase activity and regulates transcription. Mol. Cell Biol..

[53] Thompson P.R., Wang D., Wang L., Fulco M., Pediconi N., Zhang D., An W., Ge Q., Roeder R.G., Wong J., Levrero M., Sartorelli V., Cotter R.J., Cole P.A. (2004). Regulation of the p300 HAT domain *via* a novel activation loop. Nat. Struct. Mol. Biol..

[54] Marx A., Nugoor C., Muller J., Panneerselvam S., Timm T., Bilang M., Mylonas E., Svergun D.I., Mandelkow E.M., Mandelkow E. (2006). Structural variations in the catalytic and ubiquitin-associated domains of microtubule-associated protein/microtubule affinity regulating kinase (MARK) 1 and MARK2. J. Biol. Chem..

[55] Yoshiyama Y., Higuchi M., Zhang B., Huang S.M., Iwata N., Saido T.C., Maeda J., Suhara T., Trojanowski J.Q., Lee V.M. (2007). Synapse loss and microglial activation precede tangles in a P301S tauopathy mouse model. Neuron.

[56] Rouaux C., Jokic N., Mbebi C., Boutillier S., Loeffler J.P., Boutillier A.L. (2003). Critical loss of CBP/p300 histone acetylase activity by caspase-6 during neurodegeneration. EMBO J..

[57] Lu Y.N., Kavianpour S., Zhang T., Zhang X., Nguyen D., Thombre R., He L., Wang J. (2021). MARK2 phosphorylates eIF2α in response to proteotoxic stress. PLoS Biol..

[58] Timm T., Marx A., Panneerselvam S., Mandelkow E., Mandelkow E.M. (2008). Structure and regulation of MARK, a kinase involved in abnormal phosphorylation of Tau protein. BMC Neurosci..

[59] Drewes G., Ebneth A., Preuss U., Mandelkow E.M., Mandelkow E. (1997). MARK, a novel family of protein kinases that phosphorylate microtubule-associated proteins and trigger microtubule disruption. Cell.

[60] Malikov V., Naghavi M.H. (2017). Localized phosphorylation of a kinesin-1 adaptor by a capsid-associated kinase regulates HIV-1 motility and uncoating. Cell Rep..

[61] Yoshimura Y., Terabayashi T., Miki H. (2010). Par1b/MARK2 phosphorylates kinesin-like motor protein GAKIN/KIF13B to regulate axon formation. Mol. Cell Biol..

[62] Mukherjee S., Keitany G., Li Y., Wang Y., Ball H.L., Goldsmith E.J., Orth K. (2006). Yersinia YopJ acetylates and inhibits kinase activation by blocking phosphorylation. Science.

[63] Trosky J.E., Li Y., Mukherjee S., Keitany G., Ball H., Orth K. (2007). VopA inhibits ATP binding by acetylating the catalytic loop of MAPK kinases. J. Biol. Chem..

[64] Yang F.C., Tan B.C., Chen W.H., Lin Y.H., Huang J.Y., Chang H.Y., Sun H.Y., Hsu P.H., Liou G.G., Shen J., Chang C.J., Han C.C., Tsai M.D., Lee S.C. (2013). Reversible acetylation regulates salt-inducible kinase (SIK2) and its function in autophagy. J. Biol. Chem..

[65] Fenton T.R., Gwalter J., Ericsson J., Gout I.T. (2010). Histone acetyltransferases interact with and acetylate p70 ribosomal S6 kinases *in vitro* and *in vivo*. Int. J. Biochem. Cell Biol..

[66] Lan F., Cacicedo J.M., Ruderman N., Ido Y. (2008). SIRT1 modulation of the acetylation status, cytosolic localization, and activity of LKB1. Possible role in AMP-activated protein kinase activation. J. Biol. Chem..

[67] Blank M.F., Chen S., Poetz F., Schnolzer M., Voit R., Grummt I. (2017). SIRT7-dependent deacetylation of CDK9 activates RNA polymerase II transcription. Nucl. Acids Res..

[68] Augustinack J.C., Schneider A., Mandelkow E.M., Hyman B.T. (2002). Specific tau phosphorylation sites correlate with severity of neuronal cytopathology in Alzheimer's disease. Acta Neuropathol..

[69] Lund H., Gustafsson E., Svensson A., Nilsson M., Berg M., Sunnemark D., von Euler G. (2014). MARK4 and MARK3 associate with early tau phosphorylation in Alzheimer's disease granulovacuolar degeneration bodies. Acta Neuropathol. Commun..

[70] Oba T., Saito T., Asada A., Shimizu S., Iijima K.M., Ando K. (2020). Microtubule affinity-regulating kinase 4 with an Alzheimer's disease-related mutation promotes tau accumulation and exacerbates neurodegeneration. J. Biol. Chem..

[71] Rankin C.A., Sun Q., Gamblin T.C. (2007). Tau phosphorylation by GSK-3beta promotes tangle-like filament morphology. Mol. Neurodegener.

[72] Schneider A., Biernat J., von Bergen M., Mandelkow E., Mandelkow E.M. (1999). Phosphorylation that detaches tau protein from microtubules (Ser262, Ser214) also protects it against aggregation into Alzheimer paired helical filaments. Biochemistry.

