# Supplementary material for: Identification of a reciprocal negative feedback loop between tau-modifying proteins MARK2 kinase and CBP acetyltransferase
Source: J Biol Chem. 2022 Apr 22;298(6):101977. doi: 10.1016/j.jbc.2022.101977 (PMC9136110; doi:10.1016/j.jbc.2022.101977)
Supplement: Table S2.docx; APPLICATION [file mmc2.docx]

| **Construct** | **Backbone** | **Tag** | **Fragment or Mutation** | **Enzymatic activity** |
| --- | --- | --- | --- | --- |
| CBP-WT | pcDNA5/TO | FLAG | Full-length; Wild-type | **+** |
| CBP-LD | pcDNA5/TO | FLAG | Full-length; L1435A/D1436A | **−** |
| MARK2-WT | pcDNA3 | Myc | Full-length; Wild-type | **+** |
| MARK2-TE | pcDNA3 | Myc | Full-length; T208E | **+ + +** |
| MARK2-KR | pcDNA3 | Myc | Full-length; K82R | **−** |
| CBP-NES-WT | pUltra (lentiviral) | FLAG | a.a. 1088-1758; Wild-type | **+** |
| CBP-NES-LD | pUltra (lentiviral) | FLAG | a.a. 1088-1758; L1435A/D1436A | **−** |

**Table S2. A description of the gene expression plasmids used in this study.**
