# Supplementary material for: Identification of a reciprocal negative feedback loop between tau-modifying proteins MARK2 kinase and CBP acetyltransferase
Source: J Biol Chem. 2022 Apr 22;298(6):101977. doi: 10.1016/j.jbc.2022.101977 (PMC9136110; doi:10.1016/j.jbc.2022.101977)
Supplement: Figure S1.pdf; IMAGE [file mmc3.pdf]

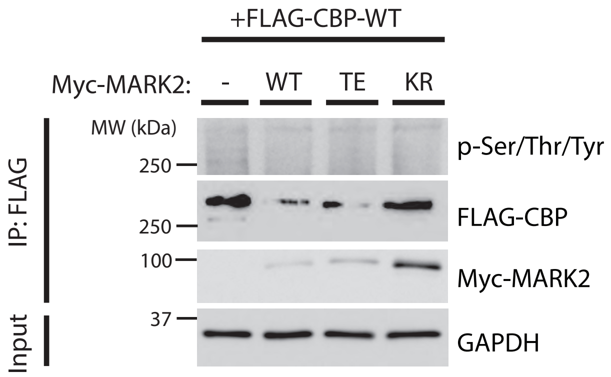

Figure S1. CBP is not detectably phosphorylated by MARK2 by phospho-blotting. To determine whether CBP is a substrate for MARK2 kinase activity, a FLAG-CBP immunoprecipitation was performed in 293A cells co-transfected with CBP and the MARK2 variants. The immunoprecipitated CBP fractions were immunoblotted with a phosphorylated Ser/Thr/Tyr antibody cocktail that detects pan-phorylated residues and no immunoreactivity was detected. GAPDH served as loading control.
